# Changing the High-Risk Behaviors of Injecting Drug Users in Iran: Application Theory of Planned Behavior

**DOI:** 10.1155/2024/4660336

**Published:** 2024-07-10

**Authors:** Ali Khani Jeihooni, Fatemeh Mohammadkhah, Mostafa Bijani, Pooyan Afzali Harsini

**Affiliations:** ^1^ Nutrition Research Center Department of Public Health School of Health Shiraz University of Medical Sciences, Shiraz, Iran; ^2^ Department of Community Health Child Nursing and Aging Ramsar School of Nursing Babol University of Medical Sciences, Babol, Iran; ^3^ Department of Medical Surgical Nursing School of Nursing Fasa University of Medical Sciences, Fasa, Iran; ^4^ Department of Public Health School of Health Kermanshah University of Medical Sciences, Kermanshah, Iran

## Abstract

**Background:**

Injection risk behavior is a major predictor of HIV infection. The present study was conducted to survey the effect of educational intervention based on the theory of planned behavior on changing high-risk behaviors (the high-risk behaviors of injecting and behaviors of transmitting blood diseases to others) of injecting drug users under the coverage of addiction harm reduction centers.

**Methods:**

This study is an experimental research on 120 drug addicts in 2021-2022. Two addiction harm reduction centers in Fasa City, Iran, were chosen randomly (one as the test group and the other as the control group). The data collection tool is made up of two parts. The first part is a questionnaire on demographics. The second part is a questionnaire based on the theory of planned behavior, which was made using information from different sources and studies. The training program was set up based on the pretest results and the theory of planned behavior for the test group. Before and six months after the educational intervention, the experimental and control groups filled out the questionnaire. With a significance level of 0.05, the independent *t*, chi-square, and paired *t* statistical tests were used to examine the data using the SPSS 22 program.

**Results:**

In the test group, the average age of addicts was 37.42 ± 10.55 years, while in the control group, the average age was 38.36 ± 10.09 years (*p*=0.244). Six months after the educational intervention, all TPB theory's constructs (knowledge, attitude, subjective norms and perceived behavioral control, behavioral intention, and behavior of injecting drug users) were higher in the test group than in the control group (*p*=0.001).

**Conclusion:**

The results show the effect of this educational intervention in reducing high-risk behaviors related to injection in injection drug addicts, so it is suggested as a useful method to reduce high-risk injection behaviors in these people.

## 1. Introduction

### 1.1. Definition of Addiction and Its Statistics

Drug addiction is defined as a chronic, relapsing disease that results from the prolonged effects of drugs on the brain. Similar to other neuropsychiatric diseases, drug addiction is intermingled with behavioral and social aspects that are equally important parts of the disease [1]. Drug abuse causes 11.8 million deaths yearly and increases the risk of lung cancer and other diseases such as heart disease, stroke, and diabetes. Additionally, alcohol and illicit drug consumption raise the risk of suicide, hepatitis, and liver illness [2]. Injecting drug use has been reported in 148 countries, of which 120 report HIV infection among this population [3]. The prevalence of drug use is higher in the Eastern Mediterranean region than global estimates, with cannabis, opium, khat, and tramadol among the main drugs used in the region [4]. The prevalence experience of drug use in Iran was 11.9% [5]. Injection drug users may be at risk of bacterial transmission of skin and soft tissue infections (SSTI) due to shared needles and injection equipment, unsuccessful injection attempts and sites, public injection, and a lack of knowledge of the correct injection site [6]. In one study in Iran, 1.6% people were HIV positive and 18.1% had a history of STIs [7]. In other study in Iran on 100 female sex workers who visited the Counseling Center for Behavioral Diseases 6 (6%) were infected with HIV, 1 (1%) with hepatitis B, and 2 (2%) were anti-HCV positive. 1 (1%) participant was suspected of having syphilis. Based on the PCR tests, 16 (16%) participants were infected with HPV [8].

### 1.2. Challenges and Solutions to Improve Injecting Behavior of Drug Addicts

To combat the opioid crisis, in Iran, the healthcare system must be strengthened. According to clinical ethics, harm reduction fosters independence, prevents harm, increases well-being, and promotes justice for drug users. According to public health ethics, harm reduction (such as injecting needle exchange training) improves health equality and cost-effectively helps vulnerable and disadvantaged groups [9]. According to WHO, among other measures to reduce the spread of AIDS in injecting drug addicts is for them to participate in preventative and safe coping programs. They facilitate access to substance abuse treatment, treatment, care, and support, as well as other important HIV-related health and welfare services (such as teaching safe syringe use, the use of disposable syringes, the proper disposal of contaminated syringes, and the availability of sterile syringes and equipment). Hygiene is required for sterile and safe injections (and opioid replacement has been introduced) [10, 11]. Furthermore, the WHO recommendations for preventing HBV and HCV transmission among injecting drug users include providing rapid HBV vaccination, providing incentives to increase uptake and completion of the HBV vaccine program, programs to provide safe needles and syringes (distribution of syringes with low dead space), and peer interventions to reduce the incidence of viral hepatitis [12]. WHO established condom distribution programs and promoted safer sexual practices through information and education as two major techniques for reducing sexually transmitted illnesses in people, particularly injecting drug addicts [13].

### 1.3. Education as One Strategy

**Figure 1:**
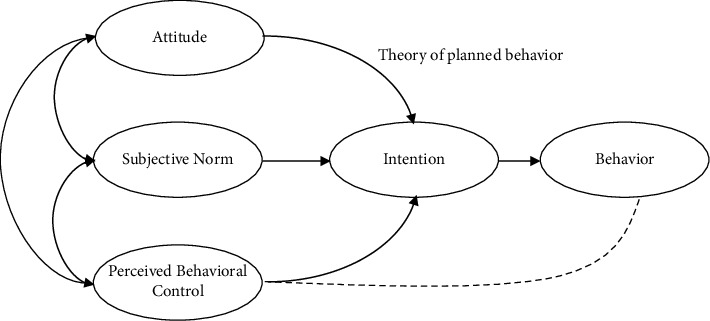
Theory of planned behavior.

Education is one strategy for preventing drug use and its effects, such as sexual implications [14]. Using theory to design and assess behavioral change treatments is currently considered useful and effective. The use of theory-based interventions has various benefits, including identifying essential structures for the target, understanding the subject, assisting in the success of the interventions, and selecting the suitable intervention approaches to utilize. The theory of planned behavior (TPB) by Ajzen and Fishbein is a famous concept that has got widespread attention in health behavior research to explain diverse health habits [15]. The elements of the TPB theory can include marijuana use among Iranian young people who seek treatment for drug use, which should be explained [16]. Theory of planned behavior that predicts the occurrence of specific behavior does, provided that the person intends to do it. According to this theory, behavioral intention (attitude towards behavior, objective norms, and perceived behavioral control) predicts intention, the direct determinant of behavior and calculated mental probability. As a result, a special behavior is formed. The concept of attitude refers to how positively or negatively a person regards the activity of interest. It necessitates considering how actions will influence outcomes. The norm of objective abstraction also expresses the refusal to confirm influential social groups such as family, friends, and colleagues finally (Figure 1). Review studies on the efficiency and effectiveness of TPB in education, psychological variables affecting different health behaviors, and sociologists also emphasized the effectiveness of TPB in describing factors affecting different environmental behaviors and different research studies. It is supported [17].

According to the findings of a study conducted by Abbas Pour and colleagues, the influence of training on the improvement of the structures of the behavioral pattern of planning in AIDS prevention behavior in addicts [18]. According to the findings of Jozaghi and colleagues' study, perceived behavioral control is one of the most critical factors in the TPB model. For injecting drug users, sharing syringes is the most predictive variable [19].

### 1.4. The Necessity and Purpose of the Study

Given the World Health Organization's emphasis on condom distribution programs and promoting safer sexual practices through information and education as two important strategies for preventing sexually transmitted diseases, including HIV, in people, particularly injecting drug users [20], the role of factors such as awareness, optimal attitude and subjective norms, in reducing the high-risk behaviors of injecting drug addicts [21–23], and the lack of research on how cognitive various health issues [24], the current study sought to survey the impact of an educational intervention based on the theory of planned behavior on modifying high-risk behaviors (the high-risk behaviors of injecting and the high-risk behaviors of transmitting blood diseases to others) of injecting drug users under the supervision of addiction harm reduction clinics.

## 2. Methods

### 2.1. Setting

In 2021–2022, the current study was an experimental intervention study. The injecting drug users who visited Fasa addiction harm reduction facilities made up the research population in this study. Two facilities from the city of Fasa's addiction harm reduction facilities were chosen randomly by coin tossing (one as the test group and the other as the control group). One hundred twenty injecting drug users were chosen for this study using the accessible sampling method (60 from each center). Being an injecting drug addict, having a history of drug abuse in addiction treatment facilities, being able to attend training sessions and counseling sessions, and providing informed consent to participate in the study were inclusion criteria. Absence from training and counseling sessions for more than one session was an exclusion criterion. Hospitalization was brought on by a mental disease diagnosis made during the course of the educational program, by death, or by migration. The subjects were guaranteed that the information gathered would be kept private after being informed of the study's goals, acquiring their consent, and getting their assent. The survey was confidential.

### 2.2. Sample Size

**Figure 2:**
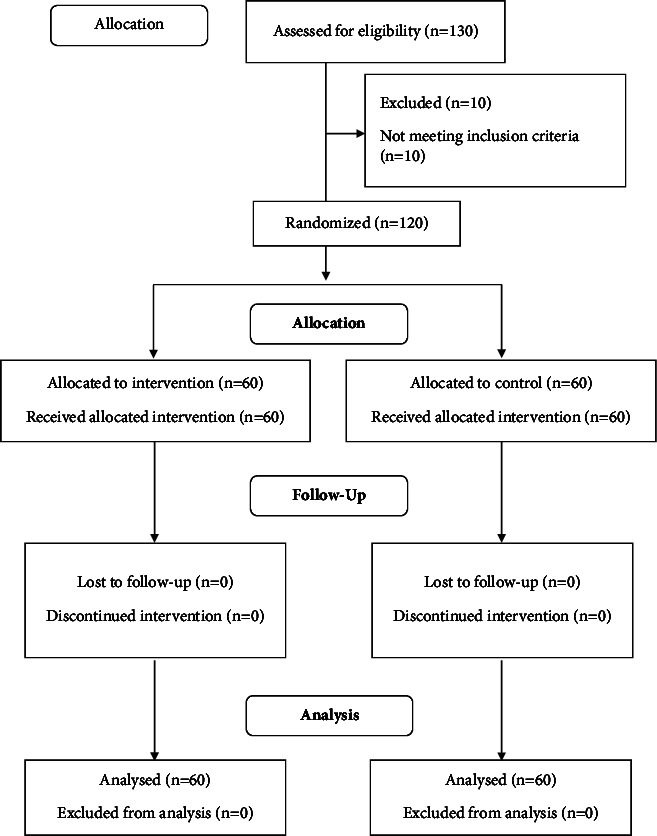
Flow diagram of the participant.

Initially, the researchers invited and enrolled 130 injecting drug users. We excluded 10 patients who did not meet the inclusion criteria; then, the remaining 120 patients were randomly allocated to an intervention (*N* = 60) and a control group (*N* = 60).  Figure 2 presents the flow diagram of the participants throughout the study (Figure 2).

### 2.3. Data Collection

The tool for gathering data consists of two parts: a demographic and background information questionnaire of people (age, education level, occupation, marital status, age of first use, duration of addiction, history of quitting, history of arrest and imprisonment, and history of drug use in the family) and a questionnaire based on the theory of planned behavior that was prepared using a variety of sources and studies [25–27]. Fifteen three-choice awareness questions (true, false, and I don't know) were part of the questionnaire for this section. Each accurate response received one point, while the incorrect and I don't know responses received 0 points. Scores for awareness ranged from 0 to 15. The following factors were measured using a Likert scale: attitude variables, subjective norms, perceived behavioral control, and behavioral intention. The scale ranged from 1 (totally disagree) to 5 (absolutely agree). Ten questions were used to assess attitude (minimum score of 10 and maximum score of 50). The minimum and highest scores for the six questions used to gauge subjective norms were 6 and 30, respectively. Ten questions were used to gauge perceived behavioral control, with a minimum score of 10 and a maximum score of 50. Fifteen questions were also included in behavioral intention (minimum score of 15 and maximum score of 75). Regarding the person's behavior, questions about risky and healthy behaviors were asked. There were 36 questions in this questionnaire (minimum score of zero and a maximum score of −36, the higher score indicates better healthy behaviors). The questionnaire's many components were made up of the following: in the first section, participants were asked general questions about their compliance with the Injury Reduction Center's training, injection behavior, including questions about altering consumption methods, injection site, number of daily injections, use of a new syringe for each injection, type of performance when a new syringe cannot be provided, how to dispose of used syringes, and whether they injected alone or with a partner. The actions that make up the behavior of spreading the illness to others include maintaining personal hygiene in public restrooms, disclosing to the hairdresser, dentist, and sex partner that one is addicted to injections, knowingly spreading the illness, administering an injection to another person, using common tools for preparing injection materials and using sharp tools. The usage of common personal goods, the history of incarceration and high-risk activities in prison, as well as the history of tattooing and cupping, during the injection time were all risk behaviors. The questionnaire also asked about family ties, smoking, changing consumer behaviors, and using the DIC health package (using sanitary packs to prevent the transmission of blood diseases caused by contaminated injections). Investigations were also conducted into the prospect of switching from injection to another type of substance use. The validity of the questionnaire's items was assessed by determining the item impact score index greater than 0.15 and the content validity ratio index higher than 0.79. Thirty injectable drug users who shared similar demographic, economic, and social characteristics with the target audience were used to target a list of prepared items to assess the tool's face validity. Twelve experts and experts (outside the study team) in the fields of health education and health promotion (9 persons), psychiatry (2 people), and psychology (1 person) were consulted to assess the content's validity. Each item was compared to the Lawshe table index, and if it was larger (0.56 for 12 persons), it was deemed required, significant, and maintained for further study. In most items, the values calculated in this study were higher than 0.70. By calculating Cronbach's alpha, the research tool's total reliability was 0.89. The questionnaire's reliability for awareness was 0.88, the attitude was 0.87, subjective norms were 0.85, perceived behavioral control was 0.82, and the behavioral intention was 0.86. The tool's reliability was assessed and found to be good because Cronbach's alpha values calculated for each of the dimensions and constructs examined in this research were more than 0.7 [28].

### 2.4. Educational Intervention

A Ph.D. in health education and health promotion, a psychiatrist, two psychologists, a social worker, and two unit experts collaborated to plan an educational program for the test group based on the results of the pretest and the theory of behavior. There was a campaign against noncommunicable diseases. The test group attended 12 sessions. Men's and women's training sessions were held separately. Group or face-to-face instruction was provided. Two 30-minute training sessions were held each week. These classes employed storytelling techniques, movie playing, slide presentations, handing out pamphlets, and displaying posters. These classes used Narcotics Anonymous (N.A.) members who recovered from addiction as trainees. In addition to providing health education, the center's health package includes syringes, condoms, distilled water, alcohol pads, and several other amenities such as food, sanitary supplies, and bathing products. It also offers free access to medical and therapeutic services, psychological counseling, social work, and other services. Addicts had access to it. Self-awareness, resilience, problem-solving, coping mechanisms, effective communication, hope and foresight, emotion management, and anger control were among the subjects covered in these sessions. One of the meetings included a family member of an addict, and the group focused on their supportive role. Individual consultations, which were held in response to the people's difficulties, requests for individual consultations, and problems, were a component of the educational interventions. Individual consultations typically lasted 60 minutes and were scheduled according to the recipient's needs. The Welfare Department representatives met in person, and their agreement was secured to provide financial assistance to those experiencing extreme poverty. People listened to the clip of the alphabet of life, which was presented to them as a PowerPoint slide show with uplifting and inspiring themes in line with the objectives of the training sessions, as well as soothing music and pictures associated with brief remarks. The control group was only invited to answer the questionnaire during a special meeting and did not receive any educational materials. The control group participated in a training session following the study's conclusion to ensure they were ethically compliant. Two experimental and control groups completed the questionnaire before the educational intervention and six months later.

### 2.5. Statistical Analysis

With a significance level of 0.05, the independent *t*, chi-square, and paired *t* statistical tests were used to examine the data using the SPSS 22 program.

## 3. Results

One hundred twenty injecting drug addicts took part in this experiment. There was no statistically significant difference between the two groups regarding the average age of addicts in the experimental group, which was 37.42 ± 10.55, and the control group, which was 38.36 ± 10.09 years old.

The chi-square test showed that the two experimental and control groups had no significant difference in terms of variables of gender, marital status, education level, occupation, age at the first use, addiction duration, quit addiction history, arrest and prison history, and history of drug use in the family (Table 1).

The findings demonstrated that, before the educational intervention, there was no discernible difference between the experimental and control groups in terms of awareness, attitude, subjective norms and perceived behavioral control, behavioral intention, and behavior. However, six months later, the experimental group demonstrated a significant increase in each of the cases above relative to the control group (Table 2).

## 4. Discussion

The study's findings revealed that following the educational intervention, the test group significantly outperformed the control group regarding knowledge, attitude, perceived behavioral control, subjective norms, behavioral intention, and change in high-risk behaviors among injecting drug users. This agrees with the findings of studies by Tyson et al. [28] and Darabi et al. [29], Malaguti et al. [30], D'Avanzo et al. [31], and Jafaraliloo et al. [32] The results of the study suggest that teaching healthy behaviors in addition to providing a suitable environment and injecting facilities can be an effective factor in reducing high-risk behaviors in injecting drug users.

The findings revealed that six months later, in the test group, the average score of people's knowledge about the preventive behaviors of high-risk behaviors of injecting drug users significantly increased. It agreed with the findings of studies by Tyson et al. [28, 33, 34]. After the educational intervention, the average scores of theoretical constructs (including awareness structure) in the intervention group significantly increased.

The experimental group demonstrated a significant increase in the mean attitude score compared to the control group six months after the educational intervention, which is consistent with the findings of the study by Keshavarzi [35], Asare et al. [36], and Tyson and his colleagues [28] that are also read. These findings showed that implementing educational intervention based on the planned behavior theory leads to increased safe behaviors in injection drug users [35]. In the current study, training sessions included lessons on attitudes and behavioral beliefs related to high-risk behaviors and the advantages of quitting, which increased participants' positive attitudes towards preventing high-risk behaviors.

The average score of perceived behavioral control in the experimental group significantly increased six months after the educational intervention. Readers can also access the findings of studies by Kenney et al. [14], Jengoué Ngamaleu et al. [37], Khalajabadi Farahani et al., [38] and Abbaspour et al. [20]. In the current study, strategies for raising perceived behavioral control of people to stop high-risk behaviors (including self-awareness training, resilience, problem-solving, coping mechanisms, effective communication, hope and foresight, emotional regulation, and anger control) were given to addicts during educational sessions, and people were asked to gradually reduce the number of risky behaviors, which has increased the perceived behavioral control score in the study.

The average score of subjective norms in the test group significantly increased six months after the educational intervention, which is consistent with the study's findings. Several other studies have been read, including Khalajabadi Farahani et al. [38], Mpeta et al. [39], Wang et al. [40], and Vatanchi et al. [41]. In the current study, a family member of an addict attended one of the meetings and stressed their supportive role, which seemed to improve the test group's score on subjective norms.

The average score for behavioral intention increased significantly in the experimental group six months after the educational intervention. In contrast, there was no significant change in the average score for behavioral intention in the control group six months later, demonstrating the impact of the educational intervention. According to research by Sadeghi et al. [34], Larki et al. [42], Keshavarzi et al. [35], Campbell et al. [43], and Mo et al. based on the idea of planned behavior on people's behavioral intentions [44]. Because education based on the theory of planned behavior boosted the test group's average scores for other TPB model structures, this study's average scores for behavioral intention have also grown [41, 42].

Additionally, three months after the educational intervention, the test group's average behavior score significantly increased. Based on the theory of planned behavior on the behavior of individuals, it demonstrates the findings from the studies by Siuki et al. [45], Darabi et al. [29], Malagut et al. [30], and Mohammadkhah et al. [46]. The likelihood of engaging in a particular behavior can be increased by having a high behavioral intention. This study's considerable reductions in the mean behavioral intention scores can therefore be considered to have significantly increased the prevention of high-risk behaviors in injecting drug users [47]. It is advised to encourage the prevention of high-risk behaviors in drug users who inject their drugs.

## 5. Limitation

This study was conducted in Fars province in the south of Iran. According to the differences in economic and social conditions between Iran and other countries, the findings of the present study might not be generalizable. Therefore, it is suggested that a similar study should be conducted in other countries. Another limitation of the current study is that there are not many studies on reducing all high-risk behaviors of injecting drug users, making it difficult to compare the findings with those studies. As a result, most studies have focused on reducing high-risk sexual behaviors in injecting drug users, and the few interventions that have attempted to reduce more risky behaviors in injecting drug addicts have not used the theory to educate participants. However, it is possible that participants may have chosen similar options for various reasons, such as social desirability bias or acquiescence bias. This factor should be considered as a limitation.

## 6. Conclusion

The results of the study showed that the use of the theory of planned behavior (TPB) has a positive effect in reducing high-risk behaviors in drug addicts; therefore, policy makers and health system managers can use this theory in predicting risky behaviors and addiction withdrawal in drug addicts. The investigation of the impact of educational intervention based on the theory of planned behavior on altering high-risk behaviors of injection drug users was made possible by the educational sessions used in this study [48].

## Figures and Tables

**Table 1:** Demographic variables of the participants (*n* = 120).

Valuable		Experimental group *N* = 60	Control group *N* = 60	*p* value
		Numbers (percent)	Numbers (percent)	
Gender	Woman	8 (13.33)	6 (10)	*p*=0/355
	Man	52 (86.67)	54 (90)	

Marital status	Single	12 (20)	10 (16.67)	*p*=0/236
	Marries	38 (63.33)	44 (73.33)	
	Divorced	8 (13.33)	6 (10)	
	Widowed	2 (3.34)	0 (0)	

Education	Illiterate	3 (5)	1 (1.67)	*p*=0/221
	Primary school	8 (13.33)	6 (10)	
	Secondary school	20 (33.33)	23 (38.33)	
	High school	28 (46.67)	30 (50)	
	College	1 (1.67)	0 (0)	

Occupation	Worker	(23.33)14	12 (20)	*p*=0/230
	Self-employed	25 (41.67)	24 (40)	
	Housewife	3 (5)	2 (3.33)	
	Unemployed	18 (30)	22 (36.67)	

Age at the first use	Under 20 years	42 (70)	38 (63.33)	*p*=0/209
	20–30 years	14 (23.33)	15 (25)	
	30–40 years	4 (6.67)	7 (11.67)	

Addiction duration	Under five years	4 (6.67)	6 (10)	*p*=0/358
	5–10 years	12 (20)	12 (20)	
	Above ten years	44 (73.33)	42 (70)	

Quit addiction history	Yes	54 (90)	51 (85)	*p*=0/348
	No	6 (10)	9 (15)	

Arrest and prison history	Yes	36 (60)	28 (46.67)	*p*=0/225
	No	24 (40)	32 (53.33)	

History of drug use in the family	Yes	39 (65)	36 (60)	*p*=0/348
	No	21 (35)	24 (40)	

**Table 2:** Comparison of the average scores of cues of the TPB model (knowledge, attitude, subjective norms and perceived behavioral control, behavioral intention, and behavior of injecting drug users) in the two test and control groups before and six months after the intervention.

Variables	Groups	Before intervention *M* ± SD	Three months after intervention *M* ± SD	*p* value
Awareness	Experimental	7/05 ± 1/12	12/10 ± 1/30	0/001
	Control	7/72 ± 1/06	8/06 ± 1/10	0/249
	*p* value	0/213	0/001	

Attitude	Experimental	21/45 ± 3/16	41/28 ± 3/47	0/001
	Control	20/88 ± 3/20	21/73 ± 3/24	0/202
	*p* value	0/242	0/001	

Subjective norms	Experimental	12/45 ± 1/33	24/81 ± 1/58	0/001
	Control	12/58 ± 1/28	12/78 ± 1/24	0/253
	*p* value	0/278	0/001	

Perceived behavioral control	Experimental	17/22 ± 3/14	43/47 ± 3/62	0/001
	Control	19/36 ± 3/19	20/38 ± 3/22	0/196
	*p* value	0/142	0/001	

Behavioral intention	Experimental	28/64 ± 4/66	60/84 ± 4/74	0/001
	Control	30/70 ± 4/72	34/67 ± 4/80	0/092
	*p* value	0/128	0/001	

Behavior	Experimental	8/14 ± 1/18	9/53 ± 1/85	0/001
	Control	9/18 ± 1/07	9/49 ± 1/13	0/246
	*p* value	0/188	0/001	

## Data Availability

The data used to support the findings of this study are available from the corresponding author upon reasonable request.

## Abbreviations

TPB: Theory of planned behavior.
